# Emerging roles and therapeutic potential of tRNA-Derived small RNAs in reproductive system diseases: a review

**DOI:** 10.3389/fcell.2025.1698265

**Published:** 2025-12-03

**Authors:** Qian Zhou, Minxin He, Min Liu, Guoying Sun, Jian Li

**Affiliations:** 1 Key Laboratory of Study and Discovery of Small Targeted Molecules of Hunan Province, Hunan Normal University H€Science Center, Changsha, Hunan, China; 2 The Engineering Research Center of Reproduction and Translational Medicine of Hunan Province, Changsha, China; 3 Key Laboratory of Model Animals and Stem Cell Biology in Hunan Province, Hunan Normal University Health Science Center, Changsha, Hunan, China; 4 Institute of Interdisciplinary Studies, Hunan Normal University, Changsha, Hunan, China; 5 Pediatric Department, Changsha Hospital for Maternal & Child Healthcare Affiliated to Hunan Normal University, Changsha, Hunan, China

**Keywords:** tsRNA, TDRs, tiRNAs, sperm, oocyte, reproductive system disease

## Abstract

tRNA-derived small RNAs (tsRNAs) are a class of non-coding RNAs(ncRNAs) generated from precursor or mature tRNAs under stress conditions, such as starvation, hypoxia, or oxidative stress. They are broadly classified into a growing class of small RNAs, known as tRNA-derived RNA (tDR), tRNA-derived small RNAs or tRNA-derived fragments and tRNA-derived stress-induced RNAs (tiRNAs) based on their cleavage sites. Recent advances in high-throughput sequencing have revealed their critical roles in the reproductive system, particularly in spermatogenesis, sperm maturation, and male infertility. In females, tsRNAs are implicated in oocyte development and embryo implantation. Dysregulation of tsRNAs has also been linked to reproductive diseases, including polycystic ovary syndrome (PCOS) and endometriosis in women, and oligospermia or azoospermia in men. Mechanistically, tsRNAs regulate gene expression, mRNA stability, and translation, influencing key pathways in reproductive health and disease. Their potential as biomarkers and therapeutic targets for reproductive disorders is increasingly recognized, though further research is needed to fully elucidate their roles and clinical applications.

## 1 Introduction

**FIGURE 1:**
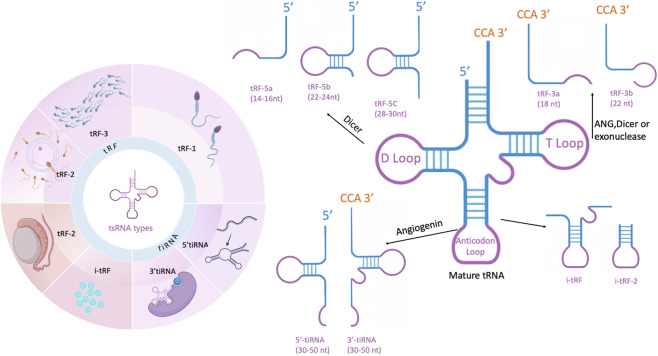
*tsRNAs derived from a wide range of mature tRNAs* (tRF-1 is produced by 3′-trailer removal from precursor tRNAs by RNase Z (ELAC2); ELAC2 generates isoforms targeted to both mitochondria and the nucleus, and thus contributes to 3′processing of mitochondrial and certain nuclear pre-tRNAs. tRF-3, arising from the 3′end of mature tRNA, is created by cleavage of the T-loop, mediated by either Dicer or angiogenin (ANG), with two distinct forms depending on where the cleavage occurs. tRF-5 is produced at the 5′end of mature tRNA through Dicer cleavage and is further divided into three categories based on its length. tRF-2 is cleaved from the anticodon loop of mature tRNA. i-tRFs mainly originate from regions between the D-loop and T-loop of the tRNA, which also include the anticodon loop. In this review, tsRNAs and tDRs are used interchangeably, with tRFs and tiRNAs representing the two major subclasses.) This figure was created with BioRender.com (License Number: *GF28ZFYV4Y*).

Reproductive system diseases, such as infertility, polycystic ovary syndrome, and endometriosis, remain significant global health challenges. Despite advancements in assisted reproductive technologies and molecular diagnostics, the underlying molecular mechanisms of these disorders are still not fully understood. Non-coding RNAs have emerged as key regulators in reproductive physiology and pathology, among which tRNA-derived small RNAs (tsRNAs) have garnered increasing attention. tsRNAs are small non-coding RNAs produced through precise cleavage from precursor or mature tRNAs (Figure 1), capable of regulating gene expression, epigenetic modifications, and intercellular communication. Recent studies suggest that tsRNAs play crucial roles in gametogenesis, embryonic development, and the transgenerational transmission of epigenetic information, highlighting their potential as biomarkers and therapeutic targets. This review aims to provide a comprehensive overview of tsRNA biogenesis, classification, mechanisms, and their functional roles in reproductive health and diseases, and proposes a conceptual roadmap for future research.

## 2 General tsRNA characteristics

### 2.1 Biogenesis and classification of tsRNAs

tRNA-derived RNAs (tDRs) represent a diverse class of small non-coding RNAs generated from precursor or mature tRNAs. In the literature, the terms “tDRs” and “tRNA-derived small RNAs (tsRNAs)” are often used interchangeably. To ensure consistency, we use tsRNAs as the primary term throughout this review, while recognizing that tDRs refer to the same group (Chen et al., 2025; Zong et al., 2021). Based on cleavage sites and biological contexts, tsRNAs can be broadly divided into two major subclasses: (i) tRNA-derived fragments (tRFs), which include several categories such as tRF-5, tRF-3, tRF-1, and internal tRFs (i-tRFs); and (ii) tRNA halves (Table 1), typically generated by cleavage at the anticodon loop under stress conditions such as oxidative stress, hypoxia, or viral infection (Xu W. et al., 2022; Kumar et al., 2014a).

**TABLE 1:** Basic Classification of tsRNAs.

Types/Names of tsRNAs	Construction	Classification	Function	References
tiRNAs	Cleavage in the D-loop or T-loop of tRNA generates short 5′-tsRNA or 3′-tsRNA.	3′tiRNA 5′tiRNA	It regulates translation by interacting with translation machinery, polysomes, processing bodies, and stress granules	Dou et al. (2019), Park et al. (2020)
tDRs	It contains three stem-loops: the D-loop, anticodon loop, and TψC loop	tRF-1 tRF-3 tRF-5 tRF-2 i-tRF	The formation of an RISC occurs through the interaction between AGO proteins and the 3′untranslated region of target mRNAs, leading to gene expression suppression. This mechanism represents a crucial post-transcriptional regulatory pathway controlling protein synthesis	Wang et al. (2013), Park et al. (2020)

The biogenesis of tsRNAs is mediated by specific ribonucleases. For example, angiogenin (ANG) and RNase T2 are known to generate tiRNAs, whereas Dicer, RNase Z, and ELAC2 contribute to the production of different tRF species. Importantly, tsRNA generation is tightly regulated by tRNA modifications (e.g., methylation, pseudouridylation) and cellular states, suggesting that tsRNAs are not random degradation products but functionally relevant small RNAs (Wang et al., 2013). As an example, 5′-tiRNAs, such as 5′-tiRNAAla and 5′-tiRNACys, with 5′-terminal oligoguanine motifs, are capable of displacing the eukaryotic translation initiation factor eIF4F from the m7GTP cap of mRNA, which results in the inhibition of translation initiation (Boskovic et al., 2020). This action leads to the formation of mRNA-protein complexes (mRNPs) and directly regulates protein translation. A growing body of research has demonstrated that tDRs and tiRNAs are significantly elevated under stress conditions, such as starvation and oxidative damage. These sRNAs regulate mRNA stability through degradation mechanisms similar to those of miRNAs. Consequently, they play critical roles in regulating key physiological processes, including apoptosis, cell proliferation, and DNA repair.

tRNA and precursor tRNA undergo cleavage to generate tsRNAs through specific pathways. A specific subclass of tRNA-derived sRNAs, known as tiRNAs, are produced primarily through cleavage of mature tRNAs under stress conditions. This cleavage occurs in the anticodon loop and is catalyzed by ANG, a secreted ribonuclease that is tightly regulated by its inhibitor RNH1 and is activated in response to oxidative stress, hypoxia, or viral infection. The role of ANG in stress-induced cleavage of tRNAs was described by Anderson (2018) and elaborated in mechanistic detail by Ivanov et al. (2011a) from the Paul Anderson laboratory, who formally introduced the concept of tiRNAs as distinct from other tRNA fragments.

tsRNAs are typically categorized into four types: 5′or 3′tRNA halves, tRF-5, tRF-3, and tRF-1. Further classification divides tsRNAs based on cleavage sites, resulting in subtypes such as tRF-1, tRF-2, tRF-3, tRF-5, tiRNA-3, and tiRNA-5 (Xu X. J. et al., 2022; Weng et al., 2022; Jianfeng et al., 2023) Their transcript lengths range from 14 to 50 nucleotides (nt), depending on where the tRNA is cleaved (Gu et al., 2022). tRF-1s arise from ELAC2/RNase Z-mediated cleavage of the 3′trailer of precursor tRNAs. Human ELAC2 (RNase Z_L) produces isoforms via alternative translation initiation that localize to both mitochondria and the nucleus/cytosol, and thus ELAC2 contributes to 3′-end processing of mitochondrial pre-tRNAs and certain nuclear-encoded pre-tRNAs (Rossmanith, 2011; Haack et al., 2013; Siira et al., 2018). tRF-3s are produced from the 3′-end of mature tRNA and are categorized into two distinct forms. tRF-5s, formed by Dicer and ANG cleavage, originate from the 5′-end of mature tRNA and terminate just before the anticodon loop, preserving the integrity of the 5′-end. These tRF-5s are further classified into three subgroups: tRF-5a, tRF-5b, and tRF-5c (Wen et al., 2021). tRF-3 is generated from the 3′-end of mature tRNA through cleavage of the T-loop by Dicer or ANG. Based on fragment length, it is divided into two variants: tRF-3a and tRF-3b. tRF-3a is produced by cleavage between nucleotides 58 and 59, resulting in an 18-nt fragment, while tRF-3b is formed by cleavage between nucleotides 54 and 55, yielding a 22-nt fragment. tRF-5 arises from the 5′-end of mature tRNA through Dicer-mediated cleavage just upstream of the anticodon loop, and exists as three isoforms (Di Fazio et al., 2022). tRF-5s are classified into three types: tRF-5a (14–16 nt), tRF-5b (22–24 nt), and tRF-5c (28–30 nt). tRF-5c is the predominant type, accounting for over 90%, with the most abundantly expressed tsRNA derived from tRNA-Gly-GCC (Ma et al., 2022a; Wang and Hu, 2022).

According to the tRF database (tRFdb), the molecular length of tRF-5 fragments typically exceeds that of tRF-3 and tRF-1 subtypes. The tRFdb database is a specialized repository that catalogs known tRNA-derived fragments across species.Among these tRNA-derived fragments, tRF-2 represents a specific category generated through the enzymatic cleavage of mature tRNA molecules at the anticodon loop region. This classification system demonstrates the structural diversity of tsRNAs based on their biogenesis pathways and cleavage positions (Wang X. Y. et al., 2023). Internal i-tRFs are produced from the central portion of mature tRNA molecules, spanning the region between the D-loop and TψC-loop, which encompasses the anticodon stem-loop structure. The biogenesis of these fragments involves the action of currently uncharacterized endonucleases. According to their 5′terminal positions, i-tRFs can be classified into three distinct subtypes: D-tRF, A-tRF, and V-tRF.Recent studies have revealed a significant correlation between the generation of both i-tRFs and tRF-2 species and cellular responses to hypoxic conditions. Despite this shared characteristic, the scientific literature often treats these tRNA-derived sRNAs as a unified category, with limited attention given to the specific roles and mechanisms of tRF-2 and i-tRFs individually.From a structural perspective, i-tRFs generally exhibit greater length compared to tRF-2 fragments. The production of both types involves specific cleavage events within the D-loop and TψC-loop regions of tRNA molecules. This shared processing pattern suggests potential similarities in their biogenesis pathways, although the precise regulatory mechanisms remain to be fully elucidated (Yang N. et al., 2024; Yu et al., 2021).Nevertheless, these tsRNAs do not reach the 5′or 3′ends. While it has been hypothesized that their length correlates with certain disease states, the precise mechanisms remain elusive.

tsRNAs are categorized based on their splice site into three groups: 5′tsRNAs, 3′tsRNAs, and a third category that includes tRF-1 and i-tRFs. The tRFdb, established in 2015, is the first dedicated tRF database (Kikuchi et al., 1990), The database contains tsRNAs from various species, including humans and mice. tRF sequences are identified by unique numbers or IDs, which facilitate the retrieval of tRF names and related experimental data (Ma et al., 2022b; Madhry et al., 2024). Other databases, such as tDRmapper, MINTbase, and tsRBase, also contribute to tsRNA research, although a unified naming convention has yet to be established (Selitsky and Sethupathy, 2015). The naming of tsRNAs should include the following components: (1) the specific classification of the tsRNA, such as tRF-1, tRF-3, 3′tiRNA, or 5′tiRNA; (2) the chromosome number; and (3) the tRNA position number. For example, tRF-3A-4-one to one refers to a tRF-3 type located at position one to one of tRNA on chromosome 4. Having outlined the classification and enzymatic generation of tsRNAs, we next focus on their diverse molecular functions and mechanisms of action, which determine their biological roles in reproduction and disease.

### 2.2 Mechanisms and functions of tsRNAs

#### 2.2.1 Post-transcriptional regulation (AGO loading, RISC assembly, miRNA-like silencing)

tRNA-derived small RNAs (tsRNAs) exert potent post-transcriptional regulatory functions by associating with Argonaute (AGO) proteins and participating in RNA-induced silencing complex (RISC) formation. Similar to miRNAs, tsRNAs guide AGO to complementary mRNA targets, leading to translational repression or mRNA degradation (Kuscu et al., 2018). For example, 5′-tRF-Gly-GCC and 5′-tRF-Glu-CTC have been shown to suppress gene expression through seed sequence complementarity within the 3′UTR of mRNAs (Liang et al., 2024). These tsRNAs fine-tune mRNA stability and translation, contributing to precise regulation of germ cell differentiation, oocyte maturation, and early embryogenesis. Beyond AGO-dependent silencing, some tsRNAs interact with RNA-binding proteins such as YBX1 and PIWI, altering mRNA stability and cellular stress responses (Shi et al., 2023). Together, these findings demonstrate that tsRNAs act as versatile regulators of post-transcriptional gene expression, integrating miRNA-like silencing and RNA–protein interactions to maintain reproductive cell homeostasis (Li D. et al., 2024; Schaefer et al., 2010).

#### 2.2.2 Translational control (ribosome interaction, translation inhibition, stress granule formation)

Beyond post-transcriptional silencing, tsRNAs directly modulate translation by interacting with ribosomal machinery. Certain 5′-tiRNAs generated under stress conditions—particularly 5′-tiRNA-Ala and 5′-tiRNA-Cys—bind to the 40S ribosomal subunit and displace eIF4G/eIF4A from mRNA caps, thereby inhibiting global protein synthesis (Ivanov et al., 2011b). This translational arrest promotes the formation of stress granules (SGs), allowing cells to conserve resources and enhance survival under oxidative or heat stress (Xie et al., 2025). In germ cells, stress-induced tsRNAs contribute to maintaining sperm quality and protecting developing gametes from damage. The reversible regulation of translation by tsRNAs reflects a crucial adaptive mechanism in both male and female reproductive systems, ensuring cellular resilience during metabolic, oxidative, or inflammatory challenges (Ma et al., 2024).

#### 2.2.3 Epigenetic inheritance and reproduction (sperm tsRNAs transmitting environmental information, follicular fluid tsRNAs as biomarkers)

tsRNAs have emerged as vital mediators of intergenerational epigenetic inheritance in reproduction. Sperm tsRNAs transmit environmental information—such as paternal diet, stress, or inflammation—to offspring, influencing embryonic metabolism and development. For instance, high-fat diet exposure alters specific sperm tsRNAs (e.g., 5′-tsRNA-Gly-GCC), which can induce metabolic disorders in progeny through changes in zygotic gene expression. Similarly, sperm tsRNAs carried by epididymosomes facilitate RNA exchange during sperm maturation, affecting sperm motility and fertilization capacity (Zhang et al., 2021; Tomar et al., 2024). In females, tsRNAs in follicular fluid and oocytes correlate with oocyte quality, ovarian reserve, and fertilization success. Distinct tsRNA signatures in follicular fluid have been proposed as potential non-invasive biomarkers for predicting assisted reproductive technology (ART) outcomes (Muraoka et al., 2024). Collectively, these findings highlight the dual roles of tsRNAs as both epigenetic transmitters and clinical indicators in reproductive health.

#### 2.2.4 Immune regulation and stress adaptation (viral replication, TLR signaling, apoptosis prevention)

Recent studies indicate that tsRNAs participate in immune modulation and cellular stress adaptation. Certain tsRNAs can inhibit viral replication by blocking reverse transcription or interfering with viral RNA translation. In addition, tsRNAs modulate innate immune signaling by binding Toll-like receptors (TLRs), thereby influencing cytokine release and inflammatory responses. For example, stress-induced 5′-tiRNAs prevent apoptosis by displacing cytochrome c from apoptosomes and promoting cell survival under oxidative stress (Xie et al., 2025; Shen et al., 2023). These immune-related roles of tsRNAs reveal an additional layer of regulation that links stress response, inflammation, and reproductive cell function, further expanding their physiological relevance beyond canonical RNA silencing (Pandey et al., 2021).

## 3 tsRNA in reproduction

tsRNAs are pivotal in epigenetic inheritance and intergenerational communication. A subclass of tsRNAs, termed tDRs, is transferred from the epididymis to sperm, transmitting paternal dietary and metabolic epigenetic information to offspring. Sperm-borne tsRNAs are highly abundant and constitute a major fraction of the small non-coding RNA population in human sperm, serving as promising biomarkers for assessing sperm quality and fertility (Sharma et al., 2016; Chen et al., 2016; Chen X. et al., 2021).Within the reproductive system, tsRNAs demonstrate significant biological activity and are particularly enriched in key reproductive components, including germ cells, developing embryos, and the maternal-fetal interface. These regulatory molecules play crucial roles in multiple reproductive processes, such as the maturation of gametes, activation of zygotic development, and regulation of early embryogenesis. Furthermore, tsRNAs serve as essential mediators in the transmission of epigenetic information across generations, contributing to the establishment and maintenance of developmental programs during critical stages of reproduction (Zhang Y. et al., 2024). Dysregulation of tsRNAs is linked to male metabolic syndrome, infertility, and intergenerational disease transmission, highlighting their role in disease etiology (Joshi et al., 2023).

Specific tsRNAs present in mature sperm play an important role in pre-implantation embryonic development (Chen et al., 2020). tsRNAs have been recognized as crucial regulators in spermatogenesis and sperm maturation, modulating mRNA stability and translation to fine-tune gene expression programs required for germ cell differentiation and sperm function (Joshi et al., 2023; Zhang Y. et al., 2024). tsRNAs display variations in their origin, sequence, length, and modifications, and their regulatory mechanisms are diverse, encompassing a broad spectrum of functions (Li Y. et al., 2024). tsRNAs, a subset of sncRNAs, play a crucial role in male fertility and the transmission of specific phenotypes across generations by impacting early-stage embryonic physiological functions in various animal models (Zhang Y. et al., 2024).Sperm tsRNAs have been shown to facilitate the intergenerational transmission of acquired traits (Zeng et al., 2021). tsRNAs in human sperm as emerging biomarkers and therapeutic candidates for male infertility (Chen X. et al., 2021). tsRNAs modulate embryonic development and subsequent phenotypic outcomes (Gong et al., 2022).

tsRNAs have garnered increasing attention in recent years for their roles in reproductive system diseases. Direct studies targeting conditions such as POI or endometriosis remain in the early stages, yet emerging evidence suggests that tsRNAs may contribute to disease pathogenesis through modulating inflammatory regulation, oxidative stress responses, and epigenetic mechanisms. In active infection or inflammatory models, the serum levels of tsRNAs are significantly elevated and correlate with the dynamics of inflammation resolution (Zhang et al., 2014). Endometriosis, a condition characterized by chronic inflammation as one of its core pathological features, suggests that tsRNAs may influence the proliferation and invasion of ectopic endometrial cells by modulating inflammatory signaling pathways. Additionally, the stability of tsRNAs in serum protein complexes positions them as promising non-invasive biomarkers for inflammation-associated diseases. tsRNAs have been implicated in cellular stress responses, with their biogenesis frequently associated with oxidative damage to tRNAs. In POI, follicular depletion is closely linked to oxidative stress, and tsRNAs may influence oocyte quality by regulating mitochondrial function or antioxidant enzyme expression. For instance, in high-fat diet-induced metabolic disorder models, sperm tsRNAs mediate transgenerational transmission of metabolic abnormalities through epigenetic mechanisms, suggesting that tsRNAs might similarly modulate the ovarian microenvironment via analogous pathways.

### 3.1 tsRNA in spermatogenesis

Spermatogenesis is a complex and highly regulated process that depends on precise gene expression control at both transcriptional and post-transcriptional levels. Increasing evidence indicates that tRNA-derived small RNAs (tsRNAs) play key regulatory roles throughout this process, extending beyond their classical function as tRNA degradation products (Sharma et al., 2016; Chen et al., 2016; Wang et al., 2025).

Recent studies have revealed that tsRNAs participate in germ cell differentiation by modulating mRNA stability and translation during spermatogonial proliferation and meiosis. For example, Sharma et al (2016) showed that specific tRNA-derived small RNAs are dynamically enriched in maturing sperm (via epididymal transfer) and can influence early embryo gene expression (Sharma et al., 2016); subsequent studies have demonstrated that some tRFs can be loaded onto Argonaute proteins and repress targets in a miRNA-like manner (e.g., Kuscu et al., 2018). Similarly, Recent studies indicate that certain 5′-tsRNAs can repress protein synthesis and modulate mRNA stability or translation—mechanisms that likely contribute to fine-tuning gene expression during spermatogonial proliferation and meiosis. For example, stress-induced 5′tRNA halves and 5′-tRFs have been shown to inhibit translation initiation or general protein synthesis (e.g., Ivanov et al., 2011a; Sobala and Hutvágner, 2013; Ivanov et al., 2011b; Sobala and Hutvagner, 2013), and other tRFs regulate mRNA stability via displacement of RNA-binding proteins (e.g., Goodarzi et al., 2015), suggesting plausible molecular routes by which tsRNAs affect spermatogenic programs. Under stress conditions, angiogenin (ANG)-mediated cleavage generates distinct 5′-tsRNAs that protect germ cells by suppressing apoptosis and promoting stress granule formation (e.g., Saikia et al., 2014). These tsRNAs serve as adaptive regulators that maintain spermatogenic cell survival and quality under oxidative or thermal stress. Moreover, Zhang *et al (*e.g., Nat Cell Biol et al., 2018) (Zhang et al., 2018) demonstrated that paternal metabolic stress alters sperm tsRNA profiles, which can influence gene expression and embryonic outcomes, underscoring their epigenetic role in reproduction.

Beyond direct gene regulation, tsRNAs also contribute to sperm maturation and intercellular communication. Altered tsRNA expression in epididymal sperm has been linked to changes in sperm motility and fertilization capacity (e.g., Wang et al., 2023; Wang H. et al., 2023). Extracellular vesicles derived from the epididymal epithelium can transfer tsRNAs to maturing sperm, shaping their final RNA composition and functional competence (e.g., Nixon et al., 2019).

Collectively, these findings establish tsRNAs as key regulators of spermatogenesis, acting through post-transcriptional silencing, translational repression, stress adaptation, and epigenetic communication. Their dynamic expression and functional diversity highlight tsRNAs as promising targets for understanding and potentially improving male fertility (Tomar et al., 2024; Zeng et al., 2021; Su et al., 2019).

### 3.2 tsRNAs in male infertility

Recent studies have demonstrated that specific sperm tsRNAs, such as tRNA-Gln-TTG fragments, are enriched in mature sperm and correlate with sperm quality and early embryo cleavage (Chen et al., 2020). Functional perturbation via antisense microinjection into human 3 PN zygotes has been shown to alter early embryonic transcriptional programs, supporting a role in early development (Wang B. et al., 2022).Environmental factors, including paternal high-fat diet or stress, can reshape sperm tsRNA populations and potentially affect offspring metabolism through epigenetic inheritance (Chen X. et al., 2021). Emerging evidence suggests that tsRNAs play pivotal roles in male fertility by regulating spermatogenesis, sperm maturation, and epididymal RNA cargo transfer.tsRNAs found in human sperm may offer new opportunities for identifying biomarkers and developing therapeutic strategies for male infertility (Tomar et al., 2024; Chen X. et al., 2021; Wang B. et al., 2022; Wu et al., 2020; Colaco and Modi, 2018). Health issues related to the male reproductive system affect the wellbeing and lifestyle of a significant number of men (Ferre-Giraldo et al., 2024). In mammals, tsRNAs are prevalent in seminal fluid and are vital for the processes of sperm maturation and successful fertilization (Han et al., 2022). Around half of infertile couples are affected by male infertility, with a significant proportion of cases classified as idiopathic (Tüttelmann et al., 2018). Earlier assumptions limited the male role in fertilization to the contribution of sperm DNA, but emerging research highlights the importance of sperm transcripts and proteins in key events like the acrosome reaction, fusion with the oocyte, and early embryo development following fertilization (Cannarella et al., 2020). For male infertility, there are three potential categories of causes: non-obstructive etiologies (issues with sperm production), obstructive etiologies (problems with the transport of sperm through the reproductive tract) (Starc et al., 2019; Jørgensen et al., 2011). It is estimated that 10%–15% of couples experience fertility issues, with approximately 50% of these cases attributed to male factors (Lei et al., 2023; Cannarella et al., 2019; Carlsen et al., 1992). Due to issues related to sperm production, one in every 20 couples is unable to conceive naturally (Nathaniel et al., 2022; Punab et al., 2017). Although semen analysis is widely regarded as the gold standard for diagnosing male infertility, its reliability is compromised by factors such as environmental influences, infections, and various pathologies that can modify the composition of semen.There is a clear need for more refined tests to assess male infertility (Yang et al., 2023a) (Table 2).

**TABLE 2:** Research on the differential expression of tsRNA in male Reproductive Diseases.

Specific disease	Key tsRNA	Biological function	Potential mechanism	References
Asthenozoospermia	tRF-5a (tRNA-Val)	Enhances sperm motility	Regulating mitochondrial function-related genes and reduces oxidative stress	
Azoospermia	tRF-Val-AAC-010	Bioinformatic analysis aims to determine the role of tsRNAs in the pathogenesis of non-obstructive azoospermia by identifying their expression patterns, potential target genes, and involvement in biological pathways. This approach helps uncover the molecular. mechanisms underlying the condition, providing insights into tsRNAs' regulatory functions and their potential as diagnostic or therapeutic targets.	Extracellular vesicle-derived tRF-Val-AAC-010 and tRF-Pro-AGG-003 have been identified as biomarkers for diagnosing non-obstructive azoospermia, offering potential for non-invasive diagnostic methods	Han et al. (2022)
Male Infertility	tsRNA	tRNA fragments can play a critical role in fertilization and embryonic development	tsRNAs are transferred to sperm during epididymal maturation, facilitating their incorporation into developing sperm cells	Joshi et al. (2023)
Prostate Cancer	tRF-1001	tRF-1001 is essential for cell proliferation and shows high expression in prostate cancer cells. Its inhibition suppresses DNA synthesis and cell growth by inducing G2 phase arrest	tiRNAs can serve as prognostic predictors for patients with prostate cancer	Jia et al. (2020)
Testicular Cancer	tiRNA (tRNA-Ile)	tRNA suppression may have an impact on healthy cells	piR-36249, a tRNA-Cys 5′fragment, forms G-quadruplexes and regulates testicular cancer progression by interacting with DHX36 to modulate OAS2 expression	Tosar (2023)

### 3.3 tsRNAs in female infertility

In oocytes and surrounding follicular cells, tsRNAs contribute to the regulation of follicular growth, oocyte maturation, and meiotic progression (Chen et al., 2020). Distinct tsRNA populations are observed in mature oocytes compared with growing follicles, suggesting stage-specific regulatory roles (Hou et al., 2024; Zhu et al., 2021). Environmental stressors such as diet or toxins can modulate tsRNA expression, potentially influencing oocyte quality and subsequent embryonic development (Saftić Martinović et al., 2024). In females, tsRNAs are implicated in oocyte quality, follicular development, and early embryonic competence.A novel category of regulatory ncRNAs, known as tsRNAs, has recently emerged as important players in cellular regulation. These molecules are produced through specific processing of transfer RNA precursors and have been implicated in numerous physiological processes, particularly in reproductive biology. Recent investigations have highlighted their significance in female reproductive health, demonstrating essential functions in multiple aspects of fertility, including the maturation process of oocytes, developmental programming of embryos, and the establishment of uterine receptivity for implantation.These molecules exhibit dynamic expression patterns during folliculogenesis and early embryogenesis, indicating their potential involvement in maintaining oocyte competence and supporting preimplantation embryo development. tsRNA are involved in premature ovarian failure (Huang et al., 2024). tsRNA profile of gestational diabetes mellitus(GDM) (Different expression patterns of specific TSRnas in GDM, indicating their association with key metabolic parameters). This highlights their good role as biomarkers for the early prediction and diagnosis of GDM. Integrating tRF into a composite biomarker group has the potential to improve clinical outcomes by enabling personalized risk assessment and targeted interventions) (Li et al., 2022); Emerging research indicates that abnormal expression patterns of tDRs and tRNA halves (tiRNAs) may play a significant role in the development of endometriosis, potentially serving as valuable biomarkers for both diagnostic and therapeutic applications. These molecular insights could substantially advance our comprehension of female infertility mechanisms while paving the way for innovative diagnostic approaches and targeted treatment strategies. Furthermore, studies have established a strong association between dysregulated tsRNA profiles and various reproductive pathologies, including PCOS and ovarian insufficiency, which represent primary causes of impaired female fertility (Devarbhavi et al., 2021). Mechanistically, tsRNAs are thought to modulate gene expression and cellular stress responses, processes that are essential for successful fertilization and embryo implantation. Notably, recent studies have proposed tsRNAs as promising biomarkers for diagnosing infertility and predicting outcomes in assisted reproductive technologies (Jia et al., 2020). Although research on tsRNAs has advanced, their specific molecular mechanisms in female reproductive health remain poorly characterized, requiring further investigation to fully understand their regulatory roles and biological functions. Further research is needed to elucidate the functional significance of tsRNAs in ovarian function, embryo quality, and endometrial receptivity. These studies have the potential to significantly enhance our comprehension of the molecular basis underlying female fertility disorders while simultaneously enabling the creation of innovative diagnostic approaches and targeted treatment strategies (Table 3).

**TABLE 3:** Research on the differential expression of tsRNA in female Reproductive Diseases.

Specific disease	Key tsRNA	Biological function	Potential mechanism	References
Premature ovarian failure	tsRNA-3043a	Enhancement of apoptosis and senescence in ovarian granulosa cells	tsRNA-3043a mimic inhibited KGN cell proliferation, induced apoptosis, increased lipid accumulation, promoted senescence, and altered the transcriptome	Huang et al. (2024)
Endometriosis	tRF/tiRNA	tRF/tiRNA expression changes in endometriosis may contribute to its pathogenesis	The RAP1 and AXON GUIDANCE pathways may drive endometriosis progression through multiple mechanisms	Li et al. (2022)
Ovarian Aging	tRF-piRNAs	The core of maintaining a youthful ovarian phenotype	tRF-piRNAs exhibit functions independent of transposon regulation in both germ cells and somatic cells, playing a role in the modulation of translation in cancer cells	Dhahbi et al. (2021)
Pregnancy Complications	5′-tRFs	The elevated secretion of placental extracellular vesicles containing 5′-tRF-Glu-CTC plays a significant role in the development and progression of preeclampsia-related pathological mechanisms	Preeclampsia-associated dysregulated 5′-tRFs can be detected in maternal plasma, with evidence pointing to a placenta-derived load	Cooke et al. (2024)
Cervical Cancer	tRF-Glu49	tRF-Glu49 suppresses cervical cell proliferation, migration, and invasion	tRF-Glu49 can regulate the proliferation, migration, and invasion of cervical cells through the FGL1 target pathway	Wang et al. (2022b)
Ovarian Cancer	tRF-03357 tRF-03358	tRF-03357 and tRF-03358 are significantly elevated in the serum of patients with high-grade ovarian cancer and in cancer cells	The tRNA-derived fragment tRF-03357 enhances malignant cell growth, motility, and invasive potential through its inhibitory effects on the tumor suppressor gene HMBOX1	Jia et al. (2020)

### 3.4 tsRNA and embroyonic development

**FIGURE 2:**
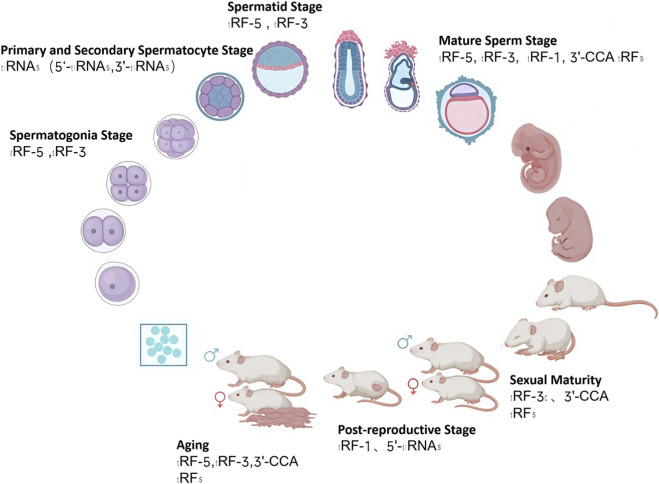
tsRNA Profile Diagram Across Mouse Developmental Stages. This figure was created with BioRender.com (License Number: *XS28ZKMHQ9)*.

During fertilization and early embryogenesis, both paternal and maternal tsRNAs play essential roles in regulating developmental gene expression. Sperm tsRNAs delivered at fertilization can influence zygotic genome activation and cleavage-stage gene regulation, while oocyte tsRNAs contribute to cytoplasmic remodeling and early developmental competence (Figure 2) (Skvortsova et al., 2018; Jianfeng et al., 2023) Experimental microinjection studies in mice have demonstrated that altered sperm tsRNA populations can recapitulate paternal diet- or stress-induced phenotypes in offspring, providing direct evidence for tsRNA-mediated epigenetic inheritance (Fan et al., 2024; Pan et al., 2021). These findings suggest that tsRNAs act as molecular messengers linking parental environmental experience to embryonic programming and offspring health (Gapp et al., 2014; Guo et al., 2021). Furthermore, tsRNA expression dynamically changes during preimplantation development and stem cell lineage specification, indicating their broader roles in cell fate determination and embryonic epigenetic regulation (Li H. M. et al., 2021).

## 4 tsRNA in reproductive diseases

Linical studies have revealed that altered tsRNA expression is associated with male infertility and impaired reproductive outcomes in humans.Several clinical studies have identified associations between specific tsRNA species and infertility-related phenotypes. For instance, sperm tRNA-Gln-TTG–derived fragments were found to correlate with sperm quality and early embryo cleavage potential; antisense disruption in human 3 PN embryos altered early transcriptional programs, suggesting functional relevance. Large-scale sperm RNA sequencing analyses also revealed distinct tsRNA expression profiles in men with idiopathic infertility compared to fertile controls, with certain tsRNA signatures predicting embryo developmental competence and IVF outcomes. In addition, tsRNAs packaged within seminal extracellular vesicles have been proposed as potential non-invasive biomarkers, as their altered abundance patterns distinguished infertile patients from normozoospermic men. Although these studies establish compelling correlations, most are observational and require validation in larger, independent cohorts (Chen X. et al., 2021; Ma et al., 2023; Hamilton et al., 2024).

Experimental studies in animal models provide causal evidence that sperm tsRNAs mediate the transmission of environmental and metabolic traits to offspring. Beyond correlative studies, several paternal exposure models in mice have demonstrated that sperm tsRNAs can exert causal effects on offspring phenotypes. For example, paternal high-fat diet or inflammatory stress reshaped sperm tsRNA populations, and microinjection of RNA fractions enriched in 5′-tsRNAs into zygotes partially reproduced offspring metabolic disorders, including altered gluconeogenesis and insulin resistance. These interventional studies provide strong evidence that sperm tsRNAs can transmit acquired metabolic information across generations. Moreover, stress-induced reprogramming of epididymal sperm tsRNA patterns has been linked to offspring behavioral and developmental outcomes, highlighting their potential role in epigenetic inheritance. Together, these findings underscore the dual nature of current evidence: human studies predominantly reveal correlations with infertility, whereas animal experiments provide mechanistic support for a causal contribution of tsRNAs to reproductive health and disease (Ma et al., 2024; Mehta and Singh, 2025).

In summary, current evidence suggests that tsRNAs are promising regulators and biomarkers in reproductive health. Human studies have mainly established correlations between altered tsRNA expression and infertility, supporting their diagnostic potential, while animal models provide stronger mechanistic evidence for their causal roles in transmitting paternal environmental and metabolic information. Future research should integrate large-scale clinical validation with functional studies to fully clarify the contribution of tsRNAs to the pathogenesis of male and female reproductive diseases.

### 4.1 Sperm tsRNAs and epigenetic regulation in male reproduction

Sperm-derived tsRNAs have emerged as critical regulators in male reproduction, exerting functions at multiple levels. First, they participate in spermatogenesis and sperm maturation by modulating germ cell development and gene expression. Second, sperm tsRNAs act as carriers of epigenetic information, transmitting paternal environmental, dietary, and metabolic influences to offspring. Finally, dysregulation of sperm tsRNAs has been implicated in male reproductive disorders, including infertility and metabolic-associated reproductive abnormalities, highlighting their potential as biomarkers and therapeutic targets.

tsRNAs are crucial for biogenesis and biological functions.Sperm tsRNAs represent paternal epigenetic factors (Chen et al., 2016; Hammoud et al., 2014). tsRNAs are involved in transgenerational inheritance (Pavelka et al., 2024). tsRNAs play a role in RNA silencing and regulating gene expression after transcription, linking them to diverse biological and disease-related functions (Ferre-Giraldo et al., 2024; Sta et al., 2018). Meanwhile, we have learned that heat stress affects the quality of sperm tsRNAs (Gan et al., 2024). tsRNAs have powerful regulatory functions (Xu et al., 2020). The biogenesis of 5′-tsRNAs in sperm mediated by ANG can lead to metabolic disorders in offspring caused by paternal inflammation (Zhang et al., 2021; Short et al., 2017). tsRNAs play a role in the intergenerational transmission of metabolically acquired traits from the paternal lineage (Hernandez et al., 2024). Paternal genetic changes can influence alterations in tsRNAs (Zhang and Hocher, 2024). Changes in tsRNA expression in small extracellular vesicles in individual semen may help identify other non-invasive biomarkers for diagnostic/prognostic purposes (Larriba et al., 2024; Swanson et al., 2020). In-depth examination of human inflammation data uncovers a relationship between shifts in epididymal sperm tsRNA patterns and later phenotypic traits (Yang et al., 2023b). Sperm tsRNA content is essential for mammalian embryo development (Chen et al., 2016; Crisóstomo et al., 2022; Donkin et al., 2016). Spermatogenesis involves the intricate transformation of male embryonic precursors into mature sperm cells within the testes (Liu et al., 2019). A single spermatogonium undergoes six consecutive mitotic divisions and two meiotic divisions to produce fully developed sperm (spermatozoa) (Yan et al., 2008). Sperm can be considered the most differentiated cells produced within any specific species (Calvel et al., 2010).

Sperm-derived tsRNAs have emerged as crucial regulators of male fertility, acting at multiple levels to control gene expression and mediate epigenetic inheritance. Generated during spermatogenesis through precise tRNA processing, sperm tsRNAs regulate post-transcriptional gene silencing and chromatin-associated pathways, thereby ensuring sperm maturation and function (Wu et al., 2024) Importantly, they also serve as paternal epigenetic factors, transmitting information about the father’s genetic background, metabolic state, and environmental exposures to offspring (Zhang and Chen, 2020).

Environmental and lifestyle factors strongly influence sperm tsRNA profiles. For example, stress, heat exposure, and high-fat diets significantly reshape tsRNA expression, with direct effects on sperm motility, quality, and fertilization potential (Zheng et al., 2021; Zhu et al., 2021) Animal studies demonstrate that altered sperm tsRNAs induced by paternal dietary or inflammatory conditions can reprogram early embryonic gene expression, predisposing offspring to metabolic disorders and other health risks. Thus, tsRNAs are not only biomarkers of sperm quality but also mediators of intergenerational inheritance (Zhang and Hocher, 2024).

Aberrant expression of sperm tsRNAs has been linked to male infertility and reproductive pathologies. Dysregulated tsRNA signatures in sperm or seminal extracellular vesicles correlate with impaired spermatogenesis, reduced semen quality, and abnormal embryonic development. Specific tsRNAs, such as Gln-TTG fragments, have shown potential as non-invasive biomarkers for assessing sperm function and predicting IVF outcomes (Wang B. et al., 2022) Beyond diagnostics, targeting tsRNA pathways may open therapeutic avenues: restoring normal tsRNA regulation could mitigate the epigenetic transmission of metabolic and reproductive disorders, offering new strategies for the treatment of male infertility (Hu et al., 2024).

In summary, sperm tsRNAs occupy a central role in male reproductive health by bridging spermatogenesis, environmental responsiveness, and epigenetic inheritance. Their dual roles as regulators of gene expression and carriers of paternal information underscore their value as both mechanistic players in reproductive biology and promising clinical tools for diagnosis and therapy.

### 4.2 The role of tsRNAs in epigenetic regulation of female reproductive diseases

tsRNAs are emerging as important epigenetic regulators in female reproductive diseases. These sRNAs, produced from tRNA processing, influence gene expression and cellular processes critical for female fertility and reproductive health. tsRNAs are involved in the regulation of key processes during oogenesis, folliculogenesis, and embryo development, thereby affecting both the quality of eggs and the success of pregnancy. tsRNAs are crucial in regulating ovarian function and follicular development, particularly in the context of follicular atresia and oocyte maturation (Huang et al., 2024). Studies have shown that tsRNAs such as tRF-5 (tRNA-Ser) modulate apoptotic pathways in aging ovaries, inducing atresia in non-viable follicles. This regulation of apoptosis is essential for maintaining the balance between follicular growth and regression, directly influencing the reproductive lifespan of females (Dhahbi et al., 2021). Disruptions in tsRNA expression can lead to impaired follicular development, contributing to reproductive disorders such as PCOS and early ovarian failure (Devarbhavi et al., 2021).

The impact of tsRNAs extends beyond the individual, influencing the transgenerational inheritanceof female reproductive health. tsRNAs have been shown to mediate the inheritance of epigenetic marks in oocytes, which can be passed down to offspring. This epigenetic regulation occurs in response to environmental factors such as diet, toxins, and stress, which alter the tsRNA profile in maternal oocytes and influence embryo development and pregnancy outcomes (Zhang and Hocher, 2024). Maternal stress and environmental factors alter tsRNA expression, linked to pregnancy complications like miscarriage and preterm birth, demonstrating their role in reproductive health inheritance. tsRNAs also regulate endometrial function and uterine receptivity during early pregnancy, influencing embryo implantation and hormonal responses (Xiong et al., 2024). tsRNAs contribute to the regulation of estrogen biosynthesis and the overall hormonal environment, which is critical for female fertility and the prevention of hormone-related diseases. Environmental factors, particularly those related to diet, pollution, and psychosocial stress, can significantly alter tsRNA profiles, leading to dysregulated gene expression in reproductive tissues. These environmental stressors may trigger epigenetic modifications that affect the function of ovarian cells, uterine cells, and the quality of eggs. Research has shown that stress-induced tsRNA dysregulation can result in altered ovarian function, leading to infertility, menstrual irregularities, and early menopause. Additionally, changes in the tsRNA profile can lead to epigenetic inheritanceof reproductive dysfunction, affecting not only the current generation but also future generations, further highlighting the importance of tsRNAs in epigenetic disease transmission. Due to their regulatory functions in gene expression and reproductive physiology, tsRNAs represent promising candidates for both diagnostic applications and therapeutic interventions in female reproductive disorders. The analysis of tsRNA patterns in oocytes, developing embryos, and endometrial tissues could provide valuable insights into reproductive health status and serve as predictive indicators for success rates in assisted reproduction procedures, particularly in IVF treatments. Moreover, targeting specific tsRNAs could offer new therapeutic strategies for managing reproductive disorders like PCOS, endometrial cancer, and infertility. By modulating the expression of key tsRNAs, it may be possible to correct epigenetic imbalances and restore normal reproductive function (Jia et al., 2020; Ma and Liu, 2022).

tsRNAs are key regulators in the epigenetic control of female reproductive health, influencing processes such as follicular development, ovarian reserve, embryo implantation, and hormonal regulation. They act as mediators of environmental effects and epigenetic inheritance, influencing both individual reproductive outcomes and those of future generations. As both diagnostic markers and therapeutic targets, tsRNAs hold great promise for improving the diagnosis and treatment of female reproductive diseases, offering new avenues for personalized reproductive medicine. Further research into the specific roles of tsRNAs in various female reproductive disorders will be critical for developing more effective and targeted therapeutic strategies.

### 4.3 tsRNAs in acquired metabolic diseases

tsRNAs play a key role in regulating pathophysiology (Chen et al., 2023). Acquired metabolic disorders refer to metabolic dysfunctions primarily caused by non-genetic factors, such as environmental influences (e.g., poor diet, sedentary lifestyle), disease states (e.g., obesity, chronic inflammation), or pharmacological interventions. These pathological conditions, encompassing type 2 diabetes mellitus, metabolic dysfunction syndrome, and non-alcoholic hepatic steatosis, are primarily defined by impaired regulation of carbohydrate metabolism, lipid processing, and cellular energy balance. Unlike inherited metabolic diseases, acquired forms often develop progressively and are closely linked to modifiable risk factors, highlighting their potential for prevention and therapeutic intervention through lifestyle modifications or targeted therapies.tsRNAs can modulate gene expression by influencing histone modifications and chromatin accessibility (Zhou et al., 2024; Zhao et al., 2021; Hnisz et al., 2015). They play a key role in regulating critical biological processes, including cancer development, metastasis, reproductive diseases, metabolic responses, and immune responses (Hnisz et al., 2015; Hlady et al., 2019; Yang P. et al., 2024). Their potential as biomarkers is an ongoing focus of research (Jia et al., 2020). tsRNAs can function through multi-target regulatory mechanisms. When multiple target genes demonstrate comparable binding potential to the same tsRNA molecule, their regulatory responses may occur in a coordinated manner, indicating the necessity for precise modulation of molecular interaction strengths (Zhang et al., 2018; Krishna et al., 2021). Obesity can also induce epigenetic changes in sperm cells (Bak et al., 2023). Alterations in tsRNA profiles could potentially function as valuable diagnostic indicators for detecting non-proliferative diabetic retinopathy among individuals with type 2 diabetes mellitus (Ding et al., 2024). Critical alterations in sperm tsRNAs may play a key role in the heritable transmission of glucose intolerance (Waldron, 2016). tsRNAs participate in numerous biological processes, encompassing post-transcriptional RNA modifications, transcriptional and translational regulation, protein biosynthesis, and fundamental cellular activities including cell cycle progression and stress adaptation mechanisms (Chu et al., 2022; Shen et al., 2018). tsRNAs participate in the regulation of vital cellular processes that are linked to the pathogenesis of major diseases, including those involved in cell proliferation, migration, and metabolism (Wu et al., 2025). There is accumulating evidence supporting the broad and persistent presence of tsRNAs across various contexts (Zhang et al., 2023). Paternal environmental factors are recognized to substantially shape various offspring phenotypes (Zhang et al., 2021). Recent findings suggest that inflammation induces ANG expression in the sperm maturation, elevating specific tRF levels in sperm. This angiogenin-driven synthesis of 5′-tsRNAs likely contributes to metabolic disturbances in offspring triggered by paternal inflammation. Current research indicates that paternal environmental experiences throughout spermatogenic development and subsequent epididymal maturation significantly influence the transmission of phenotypic characteristics to offspring through tsRNA-mediated mechanisms. These findings collectively suggest that angiogenin-mediated generation of 5′-tsRNAs in spermatozoa may contribute to the intergenerational transmission of metabolic disorders, potentially mediated by paternal inflammatory states.

The research on tsRNAs is associated with cardiac metabolic diseases (Zhang Q. et al., 2024). For a long time, these small molecules were thought to be inactive metabolic byproducts of tRNA with no function in the human body. However, recent evidence challenging this traditional view has shown that they can bind to certain target genes and influence the progression of some diseases, highlighting their indispensable role in specific biological functions. tsRNAs also hold potential therapeutic mechanisms in elderly atrial fibrillation (Luo et al., 2024). More than half of the modified tsRNA profiles originate from mitochondrial-derived tRNA precursors, demonstrating a significant correlation with impaired mitochondrial activity in cardiac pathologies associated with aging.Some compounds exert anti-aging effects by targeting mitochondria, with tsRNAs potentially serving as key epigenetic factors.

tsRNAs have emerged as significant regulatory molecules with potential implications in multiple pathological conditions, particularly in metabolic dysfunction-related diseases. However, the diagnostic relevance of plasma tsRNAs for early detection of GDM or postpartum evaluation remains largely unexplored. A recent study has examined tsRNA expression profiles in women with GDM and healthy pregnant controls across different pregnancy stages and postpartum periods, validating these findings and associating them with clinical biomarkers such as fasting blood glucose, HOMA-IR, and HbA1c. Specific tsRNAs were found to exhibit distinct expression patterns in GDM, suggesting correlations with key metabolic parameters. tsRNAs have recently been identified as important regulatory elements with significant implications across multiple disease states, particularly in conditions involving metabolic dysregulation (Hu et al., 2024). These research outcomes underscore the promising role of tsRNAs as valuable diagnostic markers for the timely identification and clinical assessment of GDM. Incorporating tDRs into composite biomarker panels could enhance clinical outcomes through personalized risk assessment and targeted therapeutic interventions.

## 5 Discussion

First, Although substantial progress has been made in elucidating the roles of tsRNAs, the field continues to grapple with several fundamental and unresolved controversies. These issues underscore the biological complexity of tsRNAs and highlight critical barriers that must be addressed to advance both mechanistic insights and translational applications—particularly in reproductive biology.One of the central controversies in the tsRNA field is whether these molecules represent functional regulatory entities or are simply byproducts of tRNA degradation. Although growing evidence supports their incorporation into ribonucleoprotein complexes and their sequence-dependent influence on mRNA translation, some argue that their accumulation may reflect passive byproducts of stress-induced cleavage rather than purposeful biogenesis. Disentangling functional relevance from stochastic degradation remains a key challenge and necessitates rigorous experimental approaches with stringent controls.

Another ongoing debate concerns the extent to which tsRNAs depend on Argonaute (AGO) (Kumar et al., 2014b) proteins to mediate gene silencing. While certain tDRs have been shown to associate with AGO1–4 and function through RISC-mediated repression, others appear to act independently of the canonical miRNA machinery—for instance, by displacing the eIF4F complex or modulating ribosome assembly directly. These findings suggest the existence of both AGO-dependent and AGO-independent pathways, highlighting the mechanistic diversity among tsRNA subtypes (Shi et al., 2019). The involvement of sperm tsRNAs in the intergenerational inheritance of acquired traits—such as those shaped by paternal diet or stress—remains both compelling and contentious (Chen et al., 2016; Sarker et al., 2019). Although studies in mouse models have revealed strong associations between altered sperm tsRNA profiles and offspring phenotypes, definitive evidence for causality is still lacking. Critical questions remain unresolved: Which specific tsRNAs are necessary and sufficient to transmit phenotypic effects? Do additional cofactors or RNA modifications mediate these outcomes? And importantly, to what extent are these mechanisms conserved in humans?

A further point of contention concerns the potential functional and mechanistic overlap between tsRNAs and other small RNAs, including miRNAs and piRNAs. Although tsRNAs share certain structural features and interact with some of the same effector proteins, their distinct biogenesis pathways and context-specific expression patterns imply unique biological roles. Nevertheless, possible cross-regulation, competitive binding, or functional redundancy may confound the interpretation of both loss-of-function and overexpression experiments. Disentangling these relationships will be critical for accurately mapping RNA-based regulatory networks (Li X. Z. et al., 2021). In addition, a practical yet consequential controversy concerns the inconsistent nomenclature and annotation of tsRNAs across commonly used databases such as tRFdb, MINTbase, and tsRBase. It is not uncommon for different studies to refer to the same tsRNA species using different names or identifiers, which complicates cross-study comparison, reproducibility, and meta-analytical efforts. Furthermore, the absence of a unified classification system impedes the integration of tsRNA datasets across species and platforms. Establishing a community-wide consensus on nomenclature and annotation is essential for ensuring clarity, consistency, and long-term progress in the field.

Second, tsRNAs have emerged as critical players in reproductive health, particularly in male infertility and related metabolic disorders. Over the past decade, studies have demonstrated that tsRNAs are abundant and stable, making them promising biomarkers for fertility assessment. Significant changes in tsRNA expression have been observed in testicular tissue, sperm, and seminal plasma of infertile men, linking specific tsRNAs to reproductive dysfunction. Beyond infertility, tsRNAs are also implicated in metabolic diseases and cancer, highlighting their broad therapeutic potential (Chen X. X. et al., 2021).tsRNAs are primarily produced in the sperm maturation and integrated into sperm during maturation, suggesting their role in post-fertilization development and transgenerational inheritance. This positions tsRNAs as key mediators of environmental influences on reproductive health. Their involvement in germ cell development, sperm maturation, and fertilization further underscores their functional importance.

Clinically, tsRNAs hold promise as diagnostic tools for sperm quality and fertility, as well as therapeutic targets for male infertility and related conditions. However, research remains in its early stages, with many questions unresolved. Future studies should focus on elucidating the precise mechanisms of tsRNAs in spermatogenesis, their regulatory networks in transgenerational inheritance, and their interactions with environmental factors. Additionally, exploring tsRNA modifications and their functional implications will be crucial for advancing their application in disease diagnosis and treatment.As an emerging field in ncRNAs research, tsRNAs present both significant opportunities and challenges. Advances in sequencing technologies and functional studies will likely uncover new tsRNAs and deepen our understanding of their biological roles. By unraveling their complex regulatory mechanisms, tsRNAs could revolutionize diagnostic and therapeutic strategies for reproductive and metabolic diseases.To date, most mechanistic insights into tDR biology have been derived from cancer or cellular stress models. In contrast, the molecular pathways by which tDRs regulate gene expression in the reproductive system—particularly in disorders such as polycystic ovary syndrome (PCOS) (Hadidi et al., 2023), premature ovarian insufficiency (POI), endometriosis, and male infertility (including azoospermia and oligozoospermia)—remain poorly characterized. Bridging this gap will require disease-specific expression profiling and functional dissection in physiologically relevant models.

tDRs hold significant promise as non-invasive biomarkers in reproductive medicine—for example, sperm-derived tDRs for assessing male fertility, and follicular fluid tDRs for evaluating ovarian reserve and function. However, their translation into clinical practice requires rigorous validation through large-scale, longitudinal studies, as well as *in vivo* functional characterization. From a therapeutic perspective, synthetic tDR mimics or antagonists may represent novel intervention strategies, though challenges in targeted delivery and molecular specificity remain substantial.In conclusion, tDR research in the context of reproduction represents a rapidly evolving yet still nascent field. Realizing its full biological and clinical potential will depend on mechanistic depth, methodological refinement, and cross-disciplinary standardization.

## 6 Conclusion and future perspectives

tRNA-derived small RNAs (tsRNAs) are increasingly recognized as versatile regulators in reproduction, influencing spermatogenesis, oocyte maturation, embryonic development, and intergenerational inheritance. Despite these advances, progress in the field remains hindered by inconsistent nomenclature, limited mechanistic insight, and variability in experimental approaches.

**FIGURE 3:**
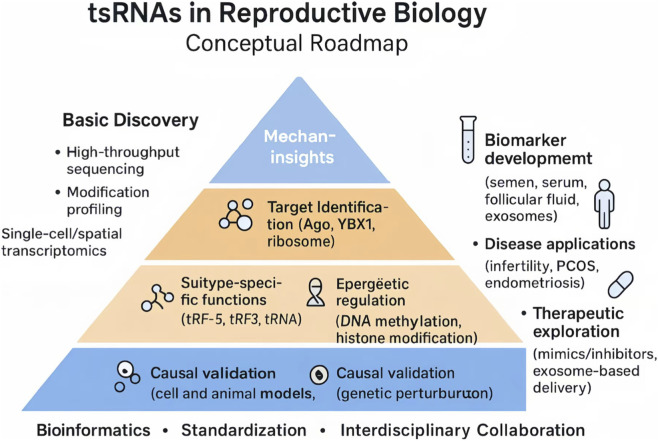
*Conceptual roadmap integrating three progressive layers:* (1) Discovery and profiling, involving high-throughput sequencing, modification mapping, and the establishment of standardized databases; (2) Mechanistic insights, including functional validation in cellular and animal models, pathway elucidation (e.g., Ago, YBX1), and causal testing using genetic perturbation; and (3) Clinical translation, encompassing biomarker development in semen, serum, or follicular fluid, disease-focused studies in infertility, PCOS, and endometriosis, as well as therapeutic exploration through synthetic mimics, inhibitors, and exosome-based delivery. These layers are horizontally supported by bioinformatics, methodological standardization, and interdisciplinary collaboration, which are indispensable for ensuring reproducibility and clinical relevance. This figure was created with BioRender.com (License Number: *ZX28ZKL0NB)*.

To address these challenges, we propose a conceptual roadmap (Figure 3) that outlines a stepwise trajectory for tsRNA research. At the discovery stage, systematic efforts should integrate high-throughput sequencing, RNA modification profiling, and single-cell or spatial transcriptomics, supported by standardized pipelines and curated databases. Mechanistic studies must move beyond correlation, focusing on subtype-specific functions (e.g., tRF-5, tRF-3, tiRNA), target identification, and the dissection of epigenetic pathways, with rigorous validation in cellular and animal models. Building on these foundations, translational research should emphasize the development of non-invasive biomarkers in reproductive fluids and tissues, explore disease-specific applications such as infertility, PCOS, and endometriosis, and evaluate therapeutic strategies using tsRNA mimics, inhibitors, and exosome-based delivery platforms.

In summary, tsRNAs represent a promising yet nascent area in reproductive medicine. Their successful translation from molecular insights to clinical applications will depend on methodological rigor, conceptual clarity, and cross-disciplinary collaboration. By following this roadmap, future studies can transform tsRNAs from emerging molecular entities into practical tools for diagnosis, prognosis, and therapy in reproductive health.

## Abbreviations

sncRNAs tsRNAs sRNAs miRNAs ANG ncRNAs, small non-coding RNAs transfer (t)RNA-derived small RNAs small RNAs microRNAs angiogenin non-coding RNAs; tiRNAs, tRNA-derived stress-induced RNAs; mRNPs, mRNA-protein complexes; mRNA, messenger RNA; tRNAs, transfer RNAs; piRNAs, Piwi-interacting RNAs; eIF4F, eukaryotic translation initiation factor 4F; m7GTP, 7-methylguanosine triphosphate; nt, nucleotide; tRFdb, tRF database; NPDR, non-proliferative diabetic retinopathy; GDM, gestational diabetes mellitus; hMSCs, Human mesenchymal stem cells; AVICs, IVF RISC Aortic Valve Interstitial Cells *In Vitro* Fertilization RNA-induced silencing complex.
